# Benefits of rapid deployment aortic valve replacement with a mini upper sternotomy

**DOI:** 10.1186/s13019-020-01268-y

**Published:** 2020-08-26

**Authors:** Siobhan Chien, Callum Clark, Saumya Maheshwari, Charilaos-Panagiotis Koutsogiannidis, Vipin Zamvar, Vincenzo Giordano, Kelvin Lim, Renzo Pessotto

**Affiliations:** 1 Department of Cardiothoracic Surgery, Royal Infirmary of Edinburgh, Edinburgh, EH16 4SA UK; 2 Department of General Medicine, University Hospital Hairmyres, East Kilbride, UK; 3 University of St Andrews, St Andrews, UK

**Keywords:** Rapid deployment aortic valve replacement, Aortic bioprosthesis, Sutureless aortic valve, Mini sternotomy, Minimal access cardiac surgery

## Abstract

**Background:**

Surgical aortic valve replacement (AVR) is currently deemed the gold standard of care for patients with severe aortic stenosis. Currently, most AVRs are safely performed through a full median sternotomy approach. With an increasingly elderly and high-risk patient population, major advances in valve technology and surgical technique have been introduced to reduce perioperative risk and post-operative complications associated with the full sternotomy approach, in order to ensure surgical AVR remains the gold standard.

For example, minimally invasive approaches (most commonly via mini sternotomy) have been developed to improve patient outcomes. The advent of rapid deployment valve technology has also been shown to improve morbidity and mortality by reducing cardiopulmonary bypass and aortic cross-clamp times, as well as facilitating the use of minimal access approaches.

Rapid deployment valves were introduced into our department at the Royal Infirmary of Edinburgh in 2014. The aim of this study is to investigate if utilising the combination of rapid deployment valves and a mini sternotomy minimally invasive approach resulted in improved outcomes in various patient subgroups.

**Methods:**

Over a 3-year period, we identified 714 patients who underwent isolated AVR in our centre. They were divided into two groups: 61 patients (8.5%) were identified who received rapid deployment AVR via J-shaped mini upper sternotomy (MIRDAVR group), whilst 653 patients (91.5%) were identified who received either a full sternotomy (using a conventional prosthesis or rapid deployment valve) or minimally invasive approach using a conventional valve (CONVAVR group). We retrospectively analysed data from our cardiac surgery database, including pre-operative demographics, intraoperative times and postoperative outcomes. Outcomes were also compared in two different subgroups: octogenarians and high-risk patients.

**Results:**

Pre-operative demographics showed that there were significantly more female and elderly patients in the MIRDAVR group. The MIRDAVR group had significantly reduced cardiopulmonary bypass (63.7 min vs. 104 min, *p* = 0.0001) and aortic cross-clamp times (47.3 min vs. 80.1 min, *p* = 0.0001) compared to the CONVAVR group. These results were particularly significant in the octogenarian population, who also had a reduced length of ICU stay (30.9 h vs. 65.6 h, *p* = 0.049). In high-risk patients (i.e. logistic EuroSCORE I > 10%), minimally invasive-rapid deployment aortic valve replacement is still beneficial and is also characterized by significantly shorter cardiopulmonary bypass time (69.1 min vs. 96.1 min, *p* = 0.03). However, post-operative correlations, such as length of ICU stay, become no more significant, likely due to serious co-morbidities in this patient group.

**Conclusion:**

We have demonstrated that minimally invasive rapid deployment aortic valve replacement is associated with significantly reduced cardiopulmonary bypass and aortic cross-clamp times. This correlation is much stronger in the octogenarian population, who were also found to have significantly reduced length of ICU stay. Our study raises the suggestion that this approach should be utilised more frequently in clinical practice, particularly in octogenarian patients.

## Background

Aortic stenosis (AS) is the commonest cause of valvular cardiac disease in developed countries, primarily caused by age-related degeneration and progressive calcification. AS is typically detected in patients aged 65 years and older [1]. Without appropriate treatment, mortality remains high in this patient subgroup, with the average survival rate only 2 years in those presenting with symptoms of cardiac failure [2].

The majority of patients therefore require aortic valve replacement (AVR). Historically, this was most commonly facilitated by surgical replacement requiring full median sternotomy [3]. Presently, AVR remains the second most common cardiac surgery performed in the United Kingdom [4]. However, demographic changes in western societies have resulted in an increasingly elderly population, with the Society of Thoracic Surgeons (STS) database highlighting that the number of patients older than 80 years has increased from 12 to 24% over the past 20 years [5]. The ageing population has triggered subsequent changes in the risk profiles of patients requiring elective or urgent AVR [6]. Major advances in valve technology and surgical technique have therefore been introduced over recent years to reduce the perioperative risk and postoperative complications associated with AVR.

For example, transcatheter aortic valve implantation (TAVI) has become increasingly popular for high-risk patients. This procedure can be undertaken using either a transapical, trans-subclavian, transcarotid or transfemoral approach, and aims to avoid the risks of sternotomy and extracorporeal circulation, as well as myocardial ischaemia due to cross-clamping of the aorta [7]. Although increasing in popularity, TAVI has associated complications including paravalvular leak, increased pacemaker rates, stroke, vascular complications and re-embolization [8]. In addition, transapical TAVI remains a surgical procedure, with similar surgical trauma when compared to minimally invasive approaches to AVR [9].

Minimally invasive approaches were introduced in an attempt to reduce the risks of full sternotomy, and were found to be associated with reduced post-operative pain and blood loss, fewer pulmonary and wound complications, and a shorter length of hospital stay [10]. The most common surgical technique utilised involves a mini-sternotomy approach, which could potentially improve post-operative recovery, shorten intensive care and hospital stay, and increase patient satisfaction overall due to superior cosmetic results [10–18]. However, these procedures are also associated with a higher grade of complexity and subsequently longer cardiopulmonary bypass (CPB) and aortic cross-clamp times [19, 20], and have therefore not become widely popular.

Rapid deployment valves (RDV) for AVR (RDAVR) have recently been introduced into clinical practice to bypass these issues. RDVs allow the utilisation of minimally invasive approaches and facilitate shorter operating times, whilst permitting surgical excision of the degenerated valve tissue [21]. The advantages of RDAVR are as follows: (1) absence or reduction for the necessity of anchoring sutures, thereby reducing the cross-clamp and extracorporeal circulation times; (2) decalcification of the annulus as well as valve implantation under direct vision to minimise paravalvular leaks by proper fitting of the prosthesis into the annulus; and (3) the possibility of performing necessary concomitant procedures, such as coronary artery bypass grafting (CABG) [22]. RDVs utilise expandable stents to enable easy and rapid implantation, thereby reducing CPB and aortic cross-clamp times, while providing excellent haemodynamic performance when compared with conventional stented valve prostheses [23–29]. The rate of valve-related complications is also low [25, 27, 29]. This highlights that the use of RDAVR may be of significant clinical benefit for patients with high operative risk, particularly when combined with a minimally invasive surgical approach [21].

In 2014, RDAVRs were introduced into our clinical practice at the Royal Infirmary of Edinburgh, thereby facilitating minimally invasive approaches to aortic valve replacement in our centre. This study aims to investigate the benefits of minimally invasive rapid deployment aortic valve replacement in various subgroups of patients within our centre.

## Methods

Over a 3-year period, we identified 714 patients who underwent isolated AVR in our centre (Royal Infirmary of Edinburgh). These patients were divided into two groups based on the implanted valve prosthesis and the surgical approach utilised.

Sixty-one patients (8.5%) were identified who received RDAVR via a J-shaped mini upper sternotomy (MIRDAVR group). Six hundred fifty-three patients (91.5%) were identified who received either a full sternotomy (using a conventional prosthesis or RDV) or minimally invasive approach using a conventional valve (CONVAVR group).

Pre-operative patient characteristics and demographics, intra-operative times and post-operative outcomes were recorded and retrospectively analysed using our departmental cardiac surgery database. Outcomes were also calculated in two different subgroups: octogenarians and high-risk patients. High-risk patients were defined as those with a pre-operative logistic EuroSCORE I of > 10%. Immediate outcomes were compared between the MIRDAVR and CONVAVR groups to determine the benefit of RDAVR using a minimal access approach.

As this study involved retrospective analysis of routinely collected data, ethical board approval was not required.

The software IBM SPSS version 24.0 was used for statistical analysis. Student’s t-test was used for two-group comparisons of continuous parametric data; the Mann–Whitney test was used for non-parametric two-group comparison. Probability values (*p* value) < 0.05 were considered statistically significant.

## Results

Data analysis of the pre-operative characteristics and demographic details showed that there were significantly more females (62.3% vs. 43.6%, *p* = 0.0001) in the MIRDAVR group, compared to the CONVAVR group. Comparison of the average age in both patient groups highlighted that there were significantly more elderly patients in the MIRDAVR group compared to the CONVAVR group (78.3 years vs. 68.9 years, *p* = 0.005). Other pre-operative characteristics were compared between the two groups, but did not reach statistical significance. This data is shown in Table 1.

**Table 1:** Pre-operative characteristics and demographics

Patient demographic	MIRDAVR (***n*** = 61)	CONVAVR (***n*** = 653)	***p*** value
**Age (years)**	78.3	68.9	0.0001
**Female gender (%)**	62.3	43.6	0.005
**Log EuroSCORE I (%)**	8.3	6.9	0.12
**NYHA IV (%)**	24.6	15.9	0.37
**LVEF < 30% (%)**	3.4	4.7	0.71
**Endocarditis (%)**	0	5.1	0.72
**Peripheral vascular disease (%)**	8.3	5.1	0.28
**Diabetes mellitus (%)**	11.5	20.8	0.08
**Hypertension (%)**	59.0	53.6	0.60
**Stroke (%)**	6.6	7.2	0.69
**COPD (%)**	13.1	14.5	0.77
**Re-do operation (%)**	1.6	5.2	0.22
**Urgency (%)**	14.8	12.9	0.61

Table 2 highlights the differences in intraoperative and postoperative outcomes between the MIRDAVR and CONVAVR groups. Notably, the MIRDAVR group had significantly shorter mean CPB times (63.7 min vs. 104.0 min, *p* = 0.0001), as well as shorter mean aortic cross-clamp times (47.3 min vs. 80.1 min, *p* = 0.0001). The MIRDAVR group also had reduced intensive care unit (ICU) stay and ventilation times compared to the CONVAVR group, however this did not reach statistical significance.

**Table 2:** Intraoperative and postoperative outcomes

Outcome	MIRDAVR (***n*** = 61)	CONVAVR (***n*** = 653)	***p*** value
**Cardiopulmonary bypass time (min)**	63.7	104.0	0.0001
**Aortic cross-clamp time (min)**	47.3	80.1	0.0001
**Ventilation (hours)**	10.7	20.1	0.18
**ICU stay (hours)**	37.2	58.7	0.15
**IABP (%)**	0	2.1	0.25
**Mortality (%)**	1.6	2.1	0.79
**Atrial fibrillation (%)**	24.6	31.1	0.29
**Permanent pacemaker (%)**	1.6	3.4	0.46
**Stroke (%)**	2.2	2.3	0.74
**Tracheostomy (%)**	0	1.2	0.39
**Haemodialysis (%)**	0	2.3	0.23
**Re-intubation (%)**	0	3.2	0.16
**Total length of stay (days)**	8.3	9.9	0.43

In octogenarians, the MIRDAVR group not only had significantly shorter CPB and aortic cross-clamp times, but also had significantly shorter length of ICU stay compared to the CONVAVR group (30.9 h vs. 65.6 h, *p* = 0.049). These results are shown in Table 3.

**Table 3:** Intraoperative and postoperative outcomes in octogenarians

Outcome	MIRDAVR (***n*** = 61)	CONVAVR (***n*** = 653)	***p*** value
**Cardiopulmonary bypass time (min)**	69.1	97.9	0.0001
**Aortic cross-clamp time (min)**	51.1	74.3	0.0001
**Ventilation (hours)**	11.7	21.2	0.082
**ICU stay (hours)**	30.9	65.6	0.049
**Total length of stay (days)**	7.8	12.2	0.059

In high risk patients (i.e. those with logistic EuroSCORE I > 10%), the MIRDAVR group had significantly shorter CPB times compared to the CONVAVR group (69.1 min vs. 96.1 min, *p* = 0.03). However, there were no significant differences in aortic cross-clamp time, length of ICU stay and ventilation times. This data is shown in Table 4.

**Table 4:** Intraoperative and postoperative outcomes in high risk patients

Outcome	MIRDAVR (***n*** = 61)	CONVAVR (***n*** = 653)	***p*** value
**Cardiopulmonary bypass time (min)**	69.1	96.1	0.03
**Aortic cross-clamp time (min)**	50.9	78.3	0.19
**Ventilation (hours)**	10.5	32.7	0.27
**ICU stay (hours)**	49.2	95.6	0.29
**Total length of stay (days)**	7.2	12.3	0.1

## Discussion

Surgical AVR has been the most commonly used procedure for treatment of aortic stenosis for many years. However, due to the growing popularity of TAVI in high-risk and elderly patients, new technologies and surgical techniques are being developed to sustain surgical AVR as the gold standard for treatment of significant aortic valve stenosis [23]. Our data correlates with this worldwide demand for the development of less risky approaches to cardiac surgery, by highlighting that elderly patients are more likely to undergo RDAVR using a minimally invasive approach. Since its inception in the early 1960s, important refinements in valve design have been made, however the basic technique of surgical valve implantation (i.e. with suture fixation to the aortic annulus) has not significantly evolved [5]. The growing popularity and success of TAVI has resulted in developments in implantation and deployment techniques, particularly aiming to facilitate the use of minimal access approaches. In particular, it has led to debate as to whether 12 to 14 sutures are genuinely essential to achieve secure valve implantation [5]. Resultantly, new techniques have been developed aiming to simplify suture technique or eliminate the use of sutures altogether by using RDVs. Benefits of using RDVs may include: (1) reduction in the time to implantation, consequently reducing the duration of myocardial ischaemia; (2) optimisation of the effective orifice area (EOA) by either eliminating the purse-string effect invariably associated with circumferential sutures or reshaping the left ventricular outflow tract to optimise flow characteristics through the bioprosthesis; and (3) facilitation of minimal access approaches resulting in reduced blood loss, shorter ICU and total hospital stays, faster recovery and enhanced cosmesis [24, 30]. However, disadvantages of RDVs include increased cost compared to a traditional surgical valve, as well as increased rate of permanent pacemaker insertion (as the fixation technique used exerts excessive radial force on the left ventricular outflow tract) [24].

The benefits of minimally invasive cardiac surgery (e.g. through mini upper sternotomy or right anterior thoracotomy) are long established, and include shorter ventilation times, reduced length of ICU and total hospital stay, and lower incidence of blood transfusion and postoperative atrial fibrillation. However, consistent disadvantages include increased CPB and cross-clamp times, possibly as this approach can make exposure and implantation of the prosthetic valve more technically challenging [31]. Prolonged CPB time and aortic cross-clamp time > 60 min have been proven to be independent predictors of morbidity and mortality in both low- and high-risk cardiac patients [32, 33]. Therefore, the synergistic effect of combining both techniques of using RDAVR with a minimal access approach maintains the above benefits, whilst combating the issue of increased CPB and aortic cross-clamp times seen with minimal access approaches alone.

These findings have been shown by the work of *Bening* et al. and *Chang* et al. Of note, these two studies highlight that the use of RDVs shorten operative times for AVR through a right anterior mini thoracotomy approach [31, 34]. Our results have also shown that these findings may be extrapolated to using RDAVR with a mini upper sternotomy approach, as we also demonstrated reduced CPB and aortic cross-clamp times in the MIRDAVR group.

These benefits were found to be much more significant in the octogenarian population in the MIRDAVR group. The European Heart Survey on valvular heart disease has shown that 33% of patients with severe symptomatic AS did not undergo surgical intervention because of an expected excessive operative risk due to advanced age or presence of significant co-morbidities [35]. Therefore, it is imperative to develop surgical strategies to prevent these patients being denied a procedure with the potential to significantly improve quality of life. Our study has shown that the octogenarians in the MIRDAVR group had significantly reduced length of ICU stay, as well as CPB and aortic cross-clamp times. This also has financial benefits for the National Health Service (NHS), with the daily cost of intensive care reported as approximately £1300, compared to £195 per day on a general hospital ward in the UK [36]. This suggests that we should be considering utilising the MIRDAVR approach more frequently in all octogenarian patients requiring isolated AVR.

However, our study is not without limitations. Firstly, this is not a randomised study and the number of patients evaluated in the MIRDAVR group is relatively small. In addition, although all patients had the same operation (isolated AVR), we compare the MIRDAVR group with a heterogeneous population (i.e. full sternotomy with conventional surgical valve; mini sternotomy with conventional surgical valve; and full sternotomy with RDV). Interestingly, this heterogeneity exists throughout all published reviews thus far [4, 21, 34], highlighting the need for a true randomised controlled trial to demonstrate these benefits. In addition, this study does not evaluate the long-term outcomes of the patients in the MIRDAVR group. Therefore, analysis of patient outcomes in this group at 1-year follow-up could provide scope for future research.

## Conclusion

We have demonstrated that surgical aortic valve replacement using a rapid deployment prosthetic valve through a minimally invasive approach is associated with significantly reduced cardiopulmonary bypass and aortic cross-clamp times, which in turn is associated with reduced morbidity and mortality. This correlation is much stronger in octogenarians, who were also found to have reduced length of ICU stay. In high risk patients, minimally invasive rapid deployment aortic valve replacement is still beneficial with reduced cardiopulmonary bypass time, although other improvements in intra- and postoperative outcomes were not found to be statistically significant in this cohort, mainly due to severe co-morbidities of these patients. Although more commonly adopted for female and elderly patients in our cohort, our study raises the possibility that we should be considering using minimally invasive rapid deployment aortic valve replacement more frequently in clinical practice, particularly in the octogenarian population.

## Data Availability

All data generated or analysed during this study are included in this published article.

## Abbreviations

AVR: Aortic valve replacement
MIRDAVR: Minimally invasive rapid deployment aortic valve replacement
CONVAVR: Conventional aortic valve replacement
ICU: Intensive care unit
AS: Aortic stenosis
STS: Society of Thoracic Surgeons
TAVI: Transcatheter aortic valve implantation
CPB: Cardiopulmonary bypass
RDV: Rapid deployment valve
RDAVR: Rapid deployment valve for aortic valve replacement
CABG: Coronary artery bypass grafting
EuroSCORE: European System for Cardiac Operative Risk Evaluation
EOA: Effective orifice area
